# Simultaneous Microendoscopic Calcium Imaging and EEG Recording of Mouse Brain during Sleep

**DOI:** 10.21769/BioProtoc.4664

**Published:** 2023-05-05

**Authors:** Sasa Teng, Yueqing Peng

**Affiliations:** 1 Institute for Genomic Medicine, Vagelos College of Physicians and Surgeons, Columbia University, New York, NY 10032, USA; 2 Department of Pathology and Cell Biology, Vagelos College of Physicians and Surgeons, Columbia University, New York, NY 10032, USA

**Keywords:** Sleep, Microendoscope, GRIN lens, EEG, Calcium imaging, Brainstem

## Abstract

Sleep is a conserved biological process in the animal kingdom. Understanding the neural mechanisms underlying sleep state transitions is a fundamental goal of neurobiology, important for the development of new treatments for insomnia and other sleep-related disorders. Yet, brain circuits controlling this process remain poorly understood. A key technique in sleep research is to monitor in vivo neuronal activity in sleep-related brain regions across different sleep states. These sleep-related regions are usually located deeply in the brain. Here, we describe technical details and protocols for in vivo calcium imaging in the brainstem of sleeping mice. In this system, sleep-related neuronal activity in the ventrolateral medulla (VLM) is measured using simultaneous microendoscopic calcium imaging and electroencephalogram (EEG) recording. By aligning calcium and EEG signals, we demonstrate that VLM glutamatergic neurons display increased activity during the transition from wakefulness to non-rapid eye movement (NREM) sleep. The protocol described here can be applied to study neuronal activity in other deep brain regions involved in REM or NREM sleep.

## Background

Sleep and wakefulness are actively controlled by the interplay of distinct neural circuits in the brain (Weber and Dan, 2016; Scammell et al., 2017). Centered on the mutual inhibition between sleep- and wakefulness-promoting circuits, a flip-flop model has been proposed to explain the mechanisms of sleep-wake state transitions (Saper et al., 2001; Saper et al., 2010). The discovery of the ascending reticular activating system (Moruzzi and Magoun, 1949), the orexin neurons (de Lecea et al., 1998; Sakurai et al., 1998), and further studies of neuromodulatory systems have greatly advanced our understanding of the neural circuits supporting wakefulness (Brown et al., 2012; Lee and Dan, 2012; Scammell et al., 2017). By contrast, the neural circuits controlling sleep have remained elusive. Several sleep-promoting regions have been identified, including the preoptic area (POA, particularly the ventrolateral preoptic area and median preoptic nucleus, or VLPO and MPO, respectively) (Sherin et al., 1996; John and Kumar, 1998; Lu et al., 2000; Alam et al., 2014; Kroeger et al., 2018), the parafacial zone (Anaclet et al., 2012 and 2014), and the basal forebrain (Xu et al., 2015). However, whether these brain structures are involved in sleep initiation or maintenance remains largely unknown. GABAergic neurons in these brain regions are sleep-active, and their activation promotes non-rapid eye movement (NREM) sleep (Scammell et al., 2017). In addition to the role of GABAergic neurons in sleep regulation, recent studies have identified glutamatergic neurons that promote NREM sleep in a few brain areas, including the perioculomotor region of the midbrain (Zhang et al., 2019), the ventrolateral periaqueductal gray of the midbrain (Zhong et al., 2019), and the posterior thalamus (Ma et al., 2019).

The POA is the most intensively studied sleep-active and sleep-promoting region. Immunohistochemistry studies in rats showed the existence of sleep-active GABAergic neurons in the VLPO and a positive correlation between the number of c-Fos-positive cells and the amount of NREM sleep (Sherin et al., 1996; Lu et al., 2002). Consistently, electrophysiological recordings demonstrated that the firing rate of VLPO sleep-active neurons correlates with the depth of NREM sleep [indicated by electroencephalogram (EEG) delta power] (Szymusiak et al., 1998; Alam et al., 2014). Notably, VLPO sleep-active neurons displayed lower activity in the wake-sleep transition period than in the subsequent sleep episode (discharge rates further increased significantly from light to deep NREM sleep) (Szymusiak et al., 1998). These findings suggest that VLPO neurons might be involved in sleep maintenance, whereas other neurons may be responsible for sleep initiation. Using c-Fos activity approach, retrograde tracing, calcium imaging, and optogenetic and chemogenetic manipulations, we recently identified a population of POA-projecting glutamatergic neurons in the ventrolateral medulla (VLM) that controls the transitions from wakefulness to NREM sleep (Teng et al., 2022).

**Figure 1.**
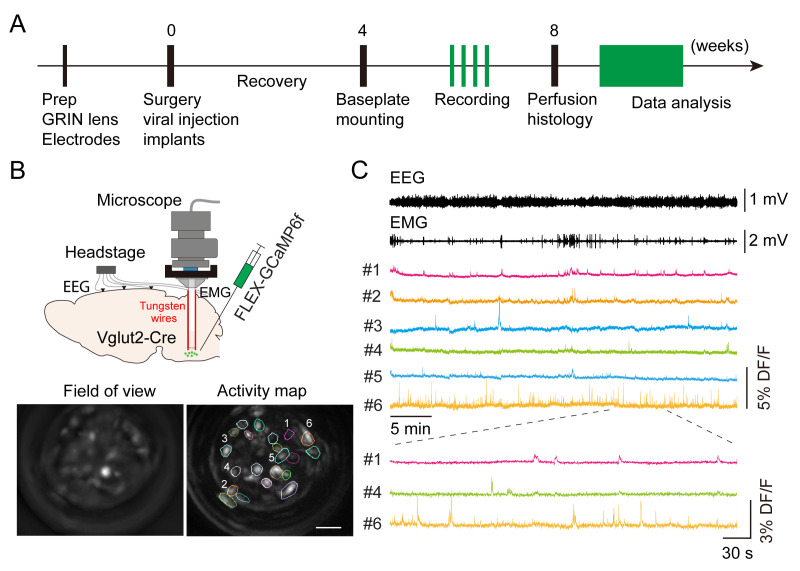
Imaging medulla glutamatergic neurons during sleep. A. Timeline of experimental design. B. Top: schematic of microendoscopic calcium imaging in the brainstem. Tungsten wires attached to the gradient refractive index (GRIN) lens were used to stabilize the field of view. Bottom: averaged GCaMP fluorescence image in the field of view and activity map of a representative imaging session in a Vglut2-Cre mouse. Scale bar, 50 μm. C. Example data showing electroencephalogram (EEG), electromyography (EMG), and representative calcium traces (DF/F) in an imaging session. Bottom: enlarged window showing calcium activity in three cells.

Here, we describe a detailed protocol for deep brain calcium imaging in the brainstem with simultaneous EEG/electromyography (EMG) recording to monitor neuronal activity during wake and sleep cycles (Figure 1). Compared to fiber photometry recording, this microendoscopic calcium imaging technique provides single-cell resolution to distinguish the function of subpopulations of VLM neurons in behaving mouse. Compared to in vivo electrophysiological recording, this imaging method allows the examination of neuronal activity in genetically defined cells. Similar in vivo calcium imaging techniques have been used to study sleep-related neuronal activity in other brain regions, including galanin-expressing GABAergic neurons in the dorsomedial hypothalamus (Chen et al., 2018) and neurotensinergic neurons in the midbrain (Zhong et al., 2019). A detailed imaging protocol in the cortical and subcortical areas has been published (Resendez et al., 2016). However, imaging the brainstem in freely moving animals typically involves significant movement artifacts. To overcome this issue, a previous study used anchoring tungsten wires glued to the gradient refractive index (GRIN) lens to stabilize the field of view (Gong et al., 2020). Here, we adopted this method to image VLM neurons in wake and sleep cycles. The whole experimental procedure takes approximately two months (Figure 1A). Our imaging protocol should be applicable to other deep brain regions that are involved in sleep regulation.

## Materials and Reagents

**Materials**

1. GRIN lens and adapter, 0.5 mm diameter, 8.4 mm length (Inscopix, catalog number: 100-004155, adapter included)
2. Base plate (Inscopix, catalog number: 100-004096)
3. Base plate cover (Inscopix, catalog number: 100-002388)
4. 5-position connectors (Mouser Electronics, catalog number: 437-8618300510001101)
5. Bone screws (J.I. Morris, catalog number: F000CE094)
6. Stainless wires, insulated, 0.003" diameter (A-M Systems, catalog number: 791100)
7. Silver wires, insulated, 0.005" diameter (A-M Systems, catalog number: 786000)
8. Flexible piano wires, 0.004" diameter (Precision Fiber Products, catalog number: SMWL-004-01)
9. Stainless steel custom-built headpost

**Reagents**

1. AAV1-syn-FLEX-GCaMP6f (Addgene, catalog number: 100833-AAV1)
2. Mineral oil (Fisher Scientific, catalog number: O121-1)
3. Contemporary Ortho-Jet^TM^ BCA powder (Lang Dental Manufacturing, catalog number: 1530BLK)
4. Ortho-Jet^TM^ liquid (Lang Dental Manufacturing, catalog number: 1304CLR)
5. Kwik-Cast silicone sealant (WPI, catalog number: KWIK-CAST)
6. 3M^TM^ Scotch-Weld epoxy adhesive DP100 clear (3M, model: DP-100)
7. Krazy glue (Krazy Glue, catalog number: KG94548R)
8. Ophthalmic ointment (Akorn, catalog number: 59399-0162-35)
9. Ketamine (Butler animal health holding, catalog number: 071069)
10. Xylazine (Butler animal health holding, catalog number: 061035)
11. Povidone iodine (MedSupply Partners, catalog number: D02-1202)

## Equipment

1. Micropipette puller (Sutter Instrument, model: P-1000)
2. Surgical microscope (Leica, model: M60)
3. Stereotaxic instruments (David Kopf Instruments, model 900-U)
4. Nanoliter 2020 injector (WPI, NANOLITER2020)
5. DC temperature controller (FHC, 40-90-8D)
6. Micro drill (WPI, model: OmniDrill35)
7. Micromanipulator (Sutter Instrument, model: MP-285A)
8. Microendoscopic calcium imaging system (Inscopix, model: nVista3)
9. Electrophysiological recording system (Neuralynx, model: Digital Lynx 4S)
10. MDF sound attenuating cubicle (Med Associates, model: ENV-018MD)
11. Custom-built head-fixation platform (Figure 2, see components below):
  - 1" XYZ translation stage (Thorlabs, catalog number: PT3)
  - Small dual-axis goniometer, 1/2" distance to point of rotation (Thorlabs, catalog number: GN2)
  - C-clamp (Siskiyou, catalog number: CC-2)
  - Post, 1/2" diameter, 8" length (Thorlabs, catalog number: TR8)
  - Parallel clamp for Ø1/2" posts, #8 counterbore and 3/16" hex (Thorlabs, catalog number: RA360)
  - Right-angle clamp for Ø1/2" posts, 3/16" hex (Thorlabs, catalog number: RA90-P5)
  - Aluminum breadboard, 6" × 12" × 1/2", 1/4"-20 taps (Thorlabs, catalog number: MB612F)
  - Mini-series aluminum breadboard, 3" × 4" × 3/8*, 8-32 and 1/4"-20 high-density taps (Thorlabs, catalog number: MSB34)
  - Custom-built metal adapter for headpost
  - M2.5 socket head cap screws for headpost
    **Figure 2.**
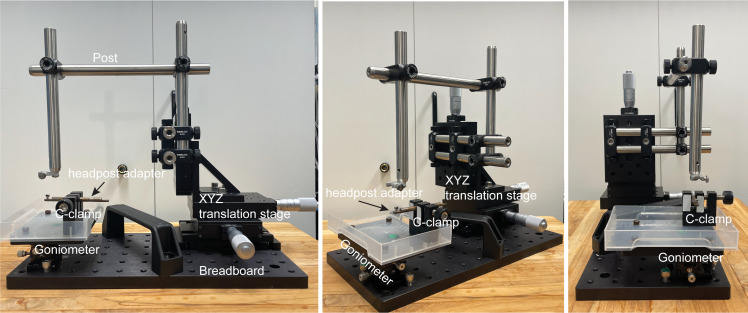
Head-fixation platform. Different views of the platform.

## Software

1. Cheetah (Neuralynx, v5.7.4)
2. MATLAB (MathWorks, version 2021)
3. IDSP software (Inscopix, IDSP 1.80)

## Procedure

- **Preparation of GRIN lens and EEG electrodes**
  1. Cut piano wires to ~5 mm length (Figure 3A–3B).
  2. Position two wires on the side of the GRIN lens, with the help of a small piece of Scotch tape (3 × 5 mm) under the microscope. The wires should be parallel to the lens, with the tips extending 200–300 μm from the GRIN lens (Figure 3C).
  3. Add 1–3 μL of Krazy glue to the wires.
  4. Wait until the Krazy glue dries.
  5. Carefully remove the Scotch tape.
  6. Keep finished GRIN lens (Figure 3D) in a container for surgical use.
  7. Cut stainless steel wires and silver wires to ~2 cm length.
  8. Solder three stainless steel wires and two silver wires to a 5-position connector. The stainless steel wires will be used as EEG electrodes, while the silver wires will be used as EMG electrodes (Figure 3E).
  9. Apply epoxy around the soldering area to secure all wires to the connector.
  10. Keep finished EEG electrodes (Figure 3E) in a container for surgical use.
  **Figure 3.**
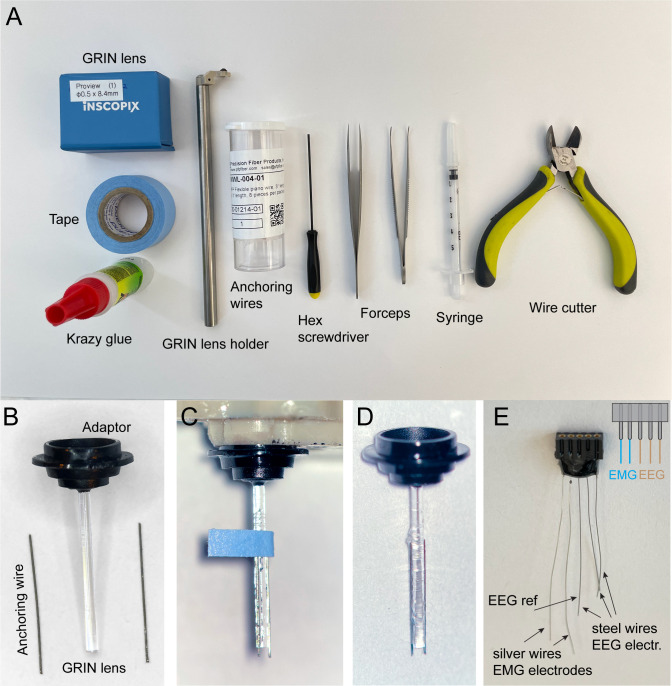
Assembled gradient refractive index (GRIN) lens and EEG electrodes. A. Tools and materials for GRIN lens preparation. B. GRIN lens and anchoring wires. C. Attachment of anchoring wires to the GRIN lens. D. Assembled GRIN lens. E. Assembled EEG/EMG electrode, inset: diagram of channel configuration. The middle wire is used as the reference (ref) for EEG.
- **Viral injection**
  1. Pull the glass capillary into a needle shape with Sutter P-1000 and load ~500 nL of AAV1-FLEX-GCaMP6f virus into the glass pipette of the nano injector.
  2. First weigh and then anesthetize mouse by intraperitoneal injection of a mixture of ketamine and xylazine (100 and 10 mg/kg).
  3. Once the mouse is anesthetized, lubricate their eyes with an application of ophthalmic ointment.
  4. Mount the animal onto a stereotaxic frame (Figure 4A) and clip the hair in a 1 × 2 cm area encircling the surgical site. Maintain normothermia by a feedback-controlled thermostatic heating pad.
  5. Disinfect the skin by swabbing three times with povidone iodine alternated with 70% alcohol, followed by a final application of povidone iodine. Then, remove the skin on the dorsal aspect of the head.
  6. Level the antero-posterior (A-P) and left-right (L-R) axis of the skull.
  7. Drill a hole of 1 mm diameter into the skull above the target coordinate (VLM, bregma -6.9 mm, lateral 1.1 mm, ventral 5.6 mm, the ventral coordinate is relative to the pial surface).
  8. Position the nano injector to the target coordinate (Figure 4B) and lower the injector slowly until its tip reaches the depth of 5.6 mm below the dorsal surface of the brain (Figure 4C). Use a low speed to minimize the damage to the surrounding brain tissues.
    **Figure 4.**
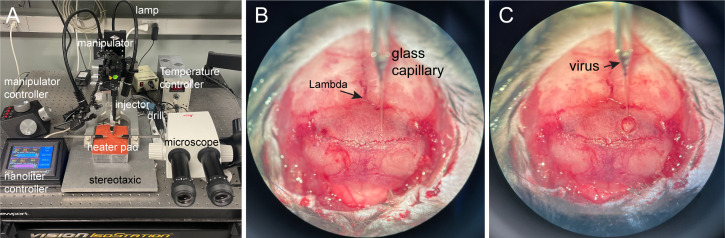
Stereotaxic viral injection. A. Equipment for viral injection. B. Positioning the nano injector to the target brain region. C. Inserting the glass capillary into the drilled brain region.
  9. Inject 200 nL of virus at a rate of 50 nL/min.
  10. Wait 5 min to allow the spread of the virus.
  11. Lift the injector slowly until the tip is out of the brain.
- **GRIN lens implantation**
  1. We used a custom-made stainless steel metal pin (400 μm in diameter, 20 mm in length, tip polished into a sharp edge) to create a path for facilitating the implantation of the GRIN lens, by inserting the metal pin 400 μm above the injection site and retracting it 2 min after the insertion (Figure 5A).
  2. Attach the GRIN lens to an Inscopix holder through the provided adapter (Figure 5B). Attach the holder on the micromanipulator.
  3. Slowly insert the GRIN lens at the speed of 10 μm/s into the brain path created by the metal pin, until it reaches 200 μm above the target coordinate (Figure 5C).
  4. Fix the GRIN lens onto the skull with the dental cement surrounding the lens (Figure 5D). Note that only the necessary amount of dental cement should be applied, to ensure most of the skull is still exposed for the following procedure. A minimum of 20 min is recommended to make sure the dental cement dried completely for the strong stabilization of the mount.
    **Figure 5.**
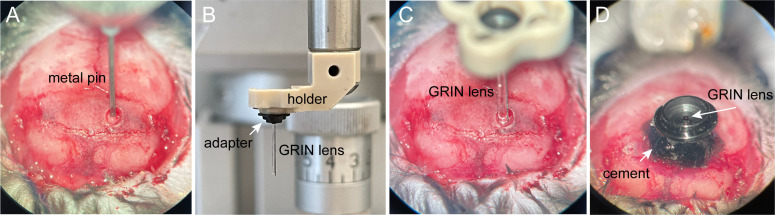
Implantation of gradient refractive index (GRIN) lens. A. Insert a metal pin to create a path for GRIN lens. B. Insert the GRIN lens. C. Secure the GRIN lens with black dental cement.
- **EEG/EMG electrodes implantation**
  1. Trim stainless steel and silver wires if needed. Remove the plastic insulation at the end of the wires with ~0.3 mm length.
  2. Drill (drill size: 0.5 mm diameter) three holes into the skull: two holes for EEG recording on top of the cortex (EEG1: 1 mm from midline, 1.5 mm anterior to the bregma; EEG2: 1 mm from midline, 1.5 mm posterior to the bregma). The third hole, for reference, should be on top of the cerebellum.
  3. Position the electrodes on top of the brain using a holder.
  4. Insert the EEG stainless wires into the holes and then secure them with bone screws. Use a screwdriver to make 2.5–3 turns for epidural position over the cortex (Figure 6A).
  5. Insert two EMG electrodes bilaterally into the neck musculature (Figure 6A).
  6. Cover the skull and implanted GRIN lens and EEG/EMG electrodes with black dental cement.
  7. Attach a metal headpost with dental cement.
  8. Cover the top of the Proview GRIN lens with silicon adhesive (Figure 6B–6C).
  9. Keep the mouse on the heat pad until recovery.
    **Figure 6.**
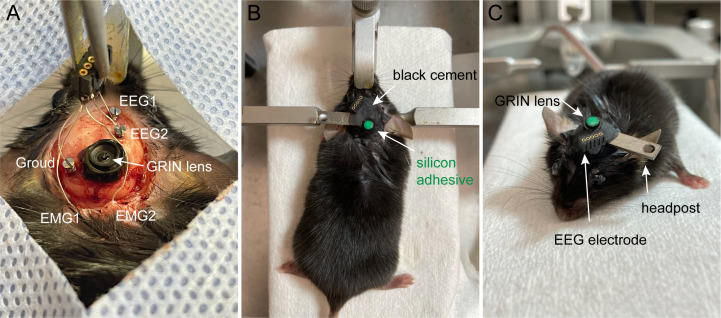
Implantation of EEG electrodes and gradient refractive index (GRIN) lens. A. Implanted EEG wires on the skull and EMG wires into the musculature. B. Cover the GRIN lens with silicon adhesive (green). C. Implants and headpost secured with black cement.
- **Mounting base plate for microendoscopic calcium imaging**
  1. After 3–4 weeks, fix the awake mouse on a custom-built head-fixation platform.
  2. Attach the baseplate to the bottom of the Inscopix miniature microendoscope.
  3. Remove the silicon cover on the GRIN lens.
  4. Position the microendoscope above the GRIN lens (Figure 7A).
  5. Adjust the angle of the head until the focal plane is parallel to the surface of the GRIN lens (Figure 7B).
  6. Find the best focal plane of the active cells (Figure 7B) by a) maximizing the number of cells in the field of view, and b) using the DF/F mode to clearly visualize real-time activity.
  7. Attach the baseplate to mouse head with dental cement (Figure 7C). Do not move the microendoscope until the dental cement is totally dry.
  8. Remove the microendoscope and cover the GRIN lens with baseplate cover (Figure 7D).
  9. Return the mouse to its home cage to rest.
    **Figure 7.**
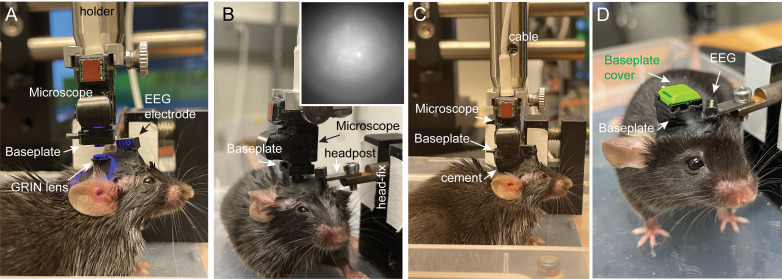
Mounting base plate for microendoscopic imaging. A. Align the baseplate with the gradient refractive index (GRIN) lens. B. Mount baseplate and microendoscope on a head-fixation platform. Inset: screenshot of a live focal plane. C. Secure the baseplate to the GRIN lens with black cement. D. EEG implants, baseplate, and baseplate cover secured with black cement.
- **Simultaneous microendoscopic calcium imaging and EEG recording**
  1. On the day of recording, connect the microendoscope and the EEG cables to the mouse on a head-fixation platform (Figure 8A).
  2. Habituate the mouse in the behavioral arena for at least 90 min.
  3. Start the imaging acquisition and sleep recording for 1–2 h as follows.
  4. Calcium activity is acquired using the nVista 3.0 hardware and IDPS software (Inscopix) with 475 nm LED illumination (10 Hz, 0.4–1.2 mW/mm^2^). EEG and EMG are recorded, bandpass filtered at 0.5–500 Hz, and digitized at 1,017 Hz using Neuralynx Digital Lynx 4S controlled by a custom-built MATLAB program via Neuralynx API.
  5. A TTL signal delivered from the Inscopix system to the Neuralynx system is used to synchronize the timing between the imaging and EEG/EMG recordings (Figure 8B).
  6. Repeat 2–3 imaging sessions to capture both wake and sleep states.
    **Figure 8.**
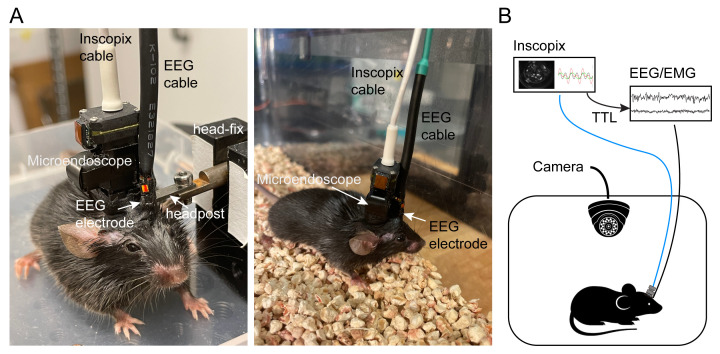
Concurrent microendoscopic calcium imaging and EEG recording. A. Left: Connect cables on a head-fixation platform. Right: Freely moving mouse with attached cables in a recording chamber. B. Schematic of recording setup.
  7. Mice are perfused for histology to examine viral expression and GRIN lens placement after recording (Figure 9).
    **Figure 9.**
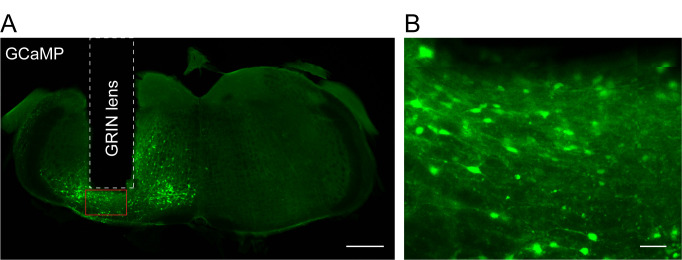
Placement of gradient refractive index (GRIN) lens and expression of GCaMP6f in the brainstem. A. Fluorescence image of a brain coronal section showing the lesion track (GRIN lens) and GCaMP6f expression. Scale bar, 0.5 mm. B. Enlarged view of the brain area under the GRIN lens (red box in A). Scale bar, 50 μm.

## Data analysis

- **Calcium imaging analysis**
  The isxd-format video file recorded during microendoscopic imaging is first processed in the IDSP software (Inscopix, IDSP 1.80). We use the default parameters in the IDSP software except those that are specifically mentioned in this protocol. Then, imaging data is further processed in MATLAB along with EEG data.
  1. Preprocessing: The maximum resolution in Inscopix nVista3 is 1,280 × 800 pixels. To speed up the whole data acquisition and analysis process, our calcium imaging was mostly recorded at the resolution of 320 × 240 pixels.
*Note: The imaging data should be spatially down-sampled to 1/4 of the original resolution if the maximum resolution is used. Use a down-sample factor of 2 if the imaging data has already been binned by 2 during image acquisition. The frame rate should be down-sampled to 10 Hz.*
  2. Spatial filter: To remove the low-frequency component (background) and the high-frequency component (noise), we process the image with the Spatial filter function in the IDSP with default bandpass filter cut-off (pixel^-1^): low 0.005, high 0.5.
  3. Motion correction: Run Motion correction function in the IDSP to minimize motion artifact between frames. Set the 100^th^ frame as the reference frame.
  4. ROI segmentation: To detect cells in the field of view, we first convert images to DF/F in the IDSP, then calculate the activity map using the max-intensity of each pixel of DF/F images in the whole imaging session. The cells in the field of view are relatively sparse, so we manually define ROIs to contain all the cells (Figure 10A).
  5. Convert raw calcium signals to DF/F: Convert the calcium signals in each ROI to DF/F in the IDSP software. The DF/F for each ROI is calculated as the difference between the calcium activity at each bin and the averaged calcium activity of the whole recording time, divided by the average (Figure 10B).
  6. The calcium traces are then deconvolved by the OASIS fast deconvolution algorithm (Friedrich et al., 2017) in MATLAB to extract calcium events (Figure 10C). Locomotion-induced artifact events are excluded by using a custom-built MATLAB program (EventReviewer, available from DOI: 10.5281/zenodo.6870710).
    **Figure 10.**
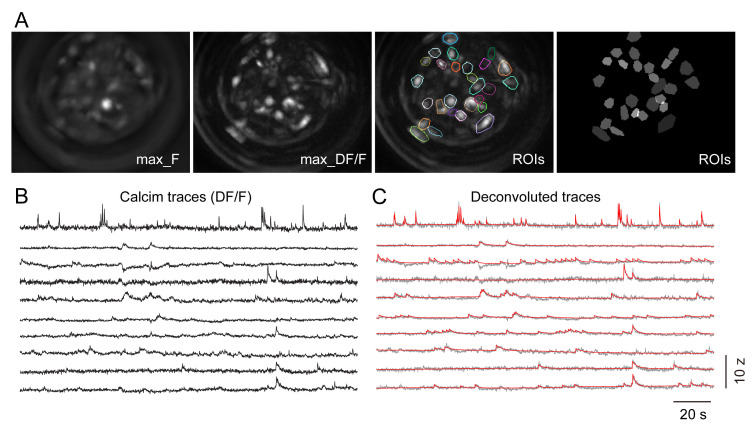
Calcium data processing. A. ROI segmentation in a recording session. Images from left to right: maximum intensity of pixels across the image session, maximum projection of fluorescent changes indicated by DF/F, DF/F image superimposed with ROI labels. Segmented ROIs for each cell. B. Calcium traces presented with Z-scored DF/F after motion-correlation. C. Deconvoluted calcium traces (red) with the OASIS algorithm.
  7. The integrated area under curve (AUC) of detected events is used for quantification.
  8. Processed data are saved as MATLAB data files.
- **EEG data analysis**
  1. Spectral analysis is carried out using fast Fourier transform (FFT) over a 5 s sliding window, sequentially shifted by 2 s increments (bins) for the whole recording session.
  2. Brain states are semi-automatically classified into wake, NREM sleep, and REM sleep states using a custom-written MATLAB program (DOI: 10.5281/zenodo.6870666) (Peng, 2022). Semi-autoclassification is validated manually by trained experimenters (Figure 11).
    **Figure 11.**
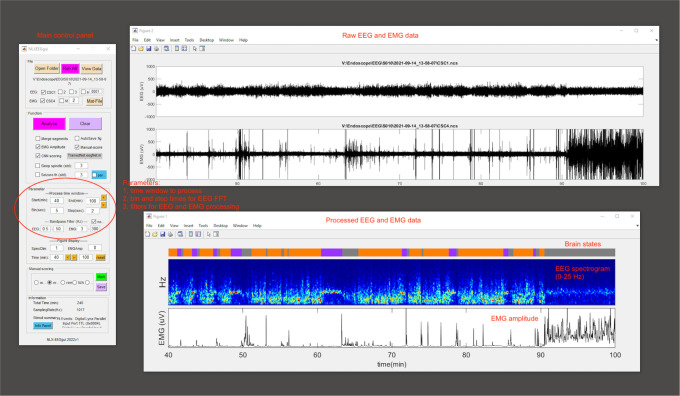
EEG data processing. A screenshot of NLXEEGgui software.
  3. The criterion for sleep/wake states are:
    Wake: desynchronized EEG and high EMG activity;
    NREM sleep: synchronized EEG with high-amplitude, delta frequency (0.5–4 Hz) activity, and low EMG activity;
    REM sleep: high power at theta frequencies (6–9 Hz) and low EMG activity.
  4. Processed EEG and EMG data are saved as MATLAB data files.
- **Alignment of microendoscopic imaging and EEG data**
  1. Load saved imaging data and EEG data into MATLAB.
  2. Align time points of imaging data and EEG data using the TTL signal in EEG data (Figure 12A and 12B).
    **Figure 12.**
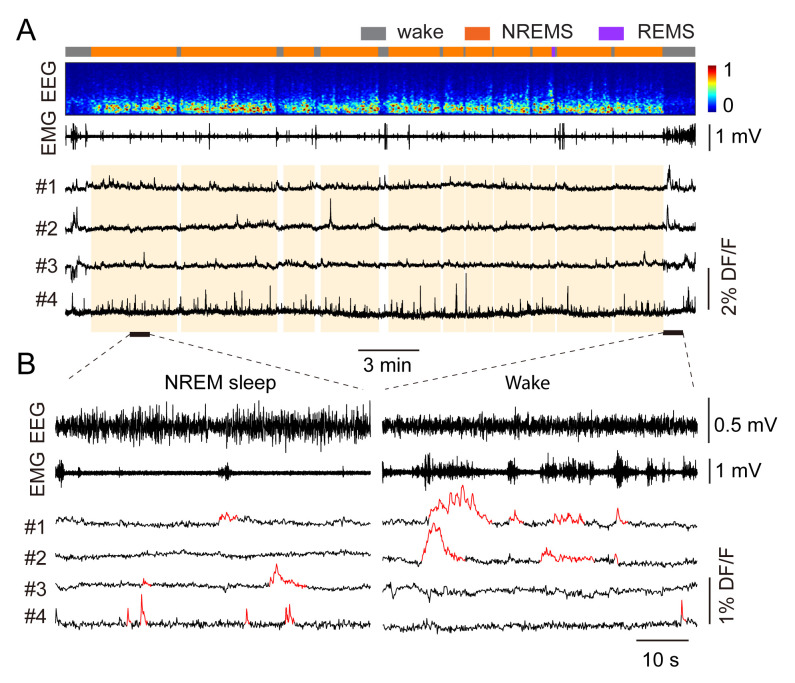
Identify sleep-related neuronal activity in the ventrolateral medulla (VLM). A. Align calcium traces with EEG data. From top to bottom: brain states (gray: wake; orange: NREM sleep; purple: REM sleep), EEG spectrogram (0–25 Hz), EMG amplitude, representative calcium traces. B. Enlarged time windows showing EEG, EMG, and calcium signals in a NREM sleep period (left) and a wake period (right). Calcium events detected by the deconvoluted algorithm are highlighted in red.
  3. To compare the activity in different brains states, we used the AUC and normalized it to the duration (in minutes) of each state, which yields relative activity per minute.
  4. We then calculated the selectivity index between brain states as:
    Index = (AUC_a_-AUC_b_)/(AUC_a_+AUC_b_)
    Where AUC_a_ and AUC_b_ refer to AUC activity in brain state a (e.g., NREM sleep) and b (e.g., wake) respectively. Index ranges from -1 to 1, with 0 indicating no selectivity between two states.
  5. To analyze calcium activity during the transitions, we calculated AUC activity in the NREM active cells before and after wake-to-NREM or NREM-to-wake transitions. A 30 s window in each brain state (e.g., 30 s wake, 30 s NREM for wake-to-NREM transitions) is used for quantification. Transitions with less than 30 s episodes in either state are excluded for analysis. Overall, we found that 57% of VLM glutamatergic cells were selectively active during wakefulness, 27% during REM sleep, and 16% during NREM sleep. Further analysis demonstrated that NREM-active neurons displayed increased activity during the transitions from wakefulness to NREM sleep, whereas their activity remains almost unchanged during the NREM-to-wake transitions. For detailed results, please refer to the published paper (Teng et al., 2022).
